# Supplementary material for: Trait paranoia shapes inter-subject synchrony in brain activity during an ambiguous social narrative
Source: Nat Commun. 2018 May 23;9:2043. doi: 10.1038/s41467-018-04387-2 (PMC5966466; doi:10.1038/s41467-018-04387-2)
Supplement: Supplementary file 1 — Supplementary Information [file 41467_2018_4387_MOESM1_ESM.pdf]

This file contains Supplementary Information for the following article:

Trait paranoia shapes inter-subject synchrony in brain activity during an ambiguous social narrative

E.S. Finn, P.R. Corlett, G. Chen, P.A. Bandettini, R.T. Constable
Nature Communications, 2018

Supplementary Note 1. Story synopsis.

Carmen is a young American doctor who journeys to the Amazon to work in a small village health clinic. There, she meets Juan, a village leader, and Alba, a young girl whom she befriends. Soon after arriving, Carmen sees a series of patients with a very serious—and seemingly highly contagious—fever. At times it seems that Juan and the villagers are fully open and forthcoming with Carmen, while at other times their behavior is harder to interpret and it seems as if they may be hiding something. Carmen begins to wonder if the villagers had known about the disease before she arrived, and if she had somehow been deliberately lured to the remote location. The story ends abruptly, when Carmen discovers that Alba herself is sick, and that unbeknownst to her, Alba is Juan's daughter. Carmen fears she may have already been infected, and wonders what to do next.

A full transcript of the story and audio recordings are available in the "stimuli" directory at the following URL: https://openneuro.org/datasets/ds001338/

Supplementary Table 1. Post-narrative multiple-choice questionnaire.

Category (total # items)	Prompt/instructions	Short name	Items*	Rating scale
Emotion (16)	"After listening to this story, I feel…"	[see list at right]	suspicious, paranoid, disturbed, confused, sad, happy, hopeful, anxious, threatened, frustrated, surprised, uncomfortable, calm, excited, inspired, amused	1 (not at all) to 5 (extremely)
Engagement, attention (2)	How engaging did you find the story?	how-engaging	(see prompt at left)	1 (very boring) to 5 (very engaging)
	How easy was it to pay attention to the story?	how-attention	(see prompt at left)	1 (very difficult) to 5 (very easy)
Character Personality Traits (3 x 6 = 18)	"Please rate Carmen/Juan/Alba on each of the following personality traits."	[character]-[trait] (e.g., carmen-trustworthy)	trustworthy, impulsive, considerate, intelligent, likeable, naïve	1 (not at all) to 5 (completely)
Character Relatability (3)	"How well could you identify with Carmen/Juan/Alba? In other words, did you find it easy or difficult to relate to her?"	[character]-identify	Carmen, Alba, Juan	1 (could not identify with her/him at all) to 5 (could identify with her/him completely)
Beliefs (6)	"Based on your beliefs about the story, rate the likelihood of each of the following scenarios."	beliefs-known	The village people had not known anything about the disease before Carmen arrived.	1 (highly unlikely) to 5 (highly likely)
		beliefs-tell	The village people didn't tell Carmen everything they knew about the disease because they thought it would scare her away.	
		beliefs-lure	Juan and other village leaders deliberately lured Carmen into a situation where they knew it was likely she would get sick.	
		beliefs-spy	Juan sent his daughter, Alba, to help cook and clean for Carmen so that he could spy/keep tabs on her.	
		beliefs-phone	Juan was being honest about why he needed to borrow Carmen's cell phone, and he had been planning to return it.	
		beliefs-sick	Juan and the other village leaders thought that having Carmen come and (potentially) get sick was the only way to get the medical help they needed.	
Predictions (2)	"In the last scene of the story, Carmen is deciding what to do. Knowing what you know about Carmen, what do you think she will do? Please pick the answer that most closely matches your beliefs."	carmen-do	(see prompt at left)	1. Carmen will go back to the village, try to treat Alba, and then stay and help any other people that come down with the sickness.<br>2. Carmen will go back to the village, but only to try to treat Alba. Once Alba is cured (or if Alba dies), she will leave right away.<br>3. Carmen will escape, but she will not tell anyone that she was suspicious of the village people. She will send them outside help.<br>4. Carmen will escape, and tell everyone that she is suspicious of the village people and thinks she was tricked into going there.
	"If you were in Carmen's situation, what would you do? Please pick the answer that most closely matches your beliefs."	you-do	(see prompt at left)	1. I would go back to the village and continue helping the people that got sick.<br>2. I would go back, but only to try to help Alba. Then I would leave immediately.<br>3. I would escape right away, but I wouldn't tell anyone that I was suspicious of the village people. I would call for help to be sent to them.<br>4. I would escape right away and tell people that I was suspicious, and I thought I had been tricked into going to the village.

*For the Emotion, Character Personality Traits, and Beliefs categories, the order in which the items were presented was randomized across participants.

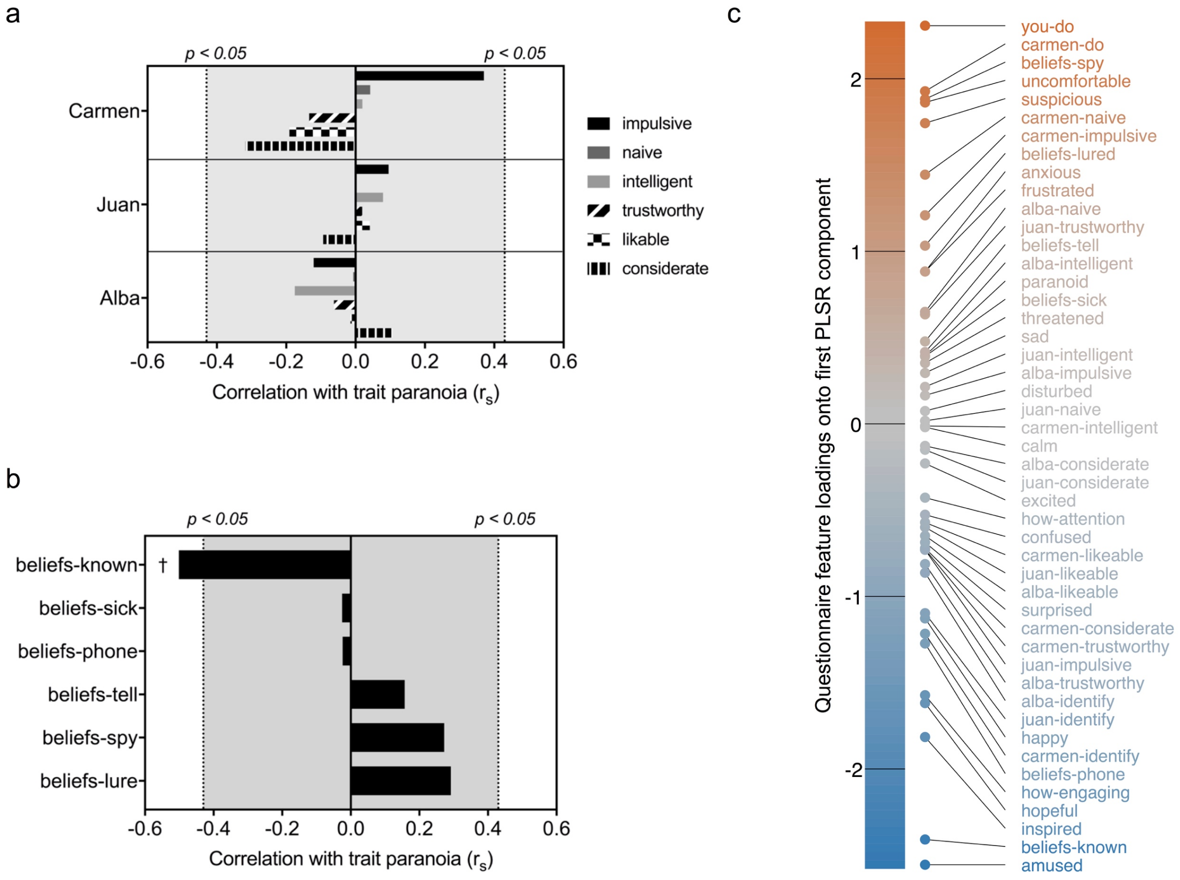

Supplementary Fig. 1. Relationship between multiple-choice questionnaire items and trait paranoia. a) Rank correlations between participants' trait paranoia score and their ratings of each of the three principal characters on six personality traits (based on a Likert scale from 1 to 5). No correlations were significant at $p < 0.05$ (uncorrected). b) Rank correlations between participants' trait paranoia score and their likelihood ratings of various potential scenarios ("beliefs") following the narrative (based on a Likert scale from 1 to 5). Likelihood rating for one scenario (denoted with †) was significant at $p < 0.05$ but this did not survive correction for multiple comparisons. c) Loadings of all multiple-choice items for the first component from a partial least squares regression relating features of the multiple-choice questionnaire to trait paranoia score, sorted by strength and direction of association with paranoia (those positively related to paranoia at top in orange; those inversely related at bottom in blue). See Supplementary Table 1 for a dictionary of all abbreviations and short item names.

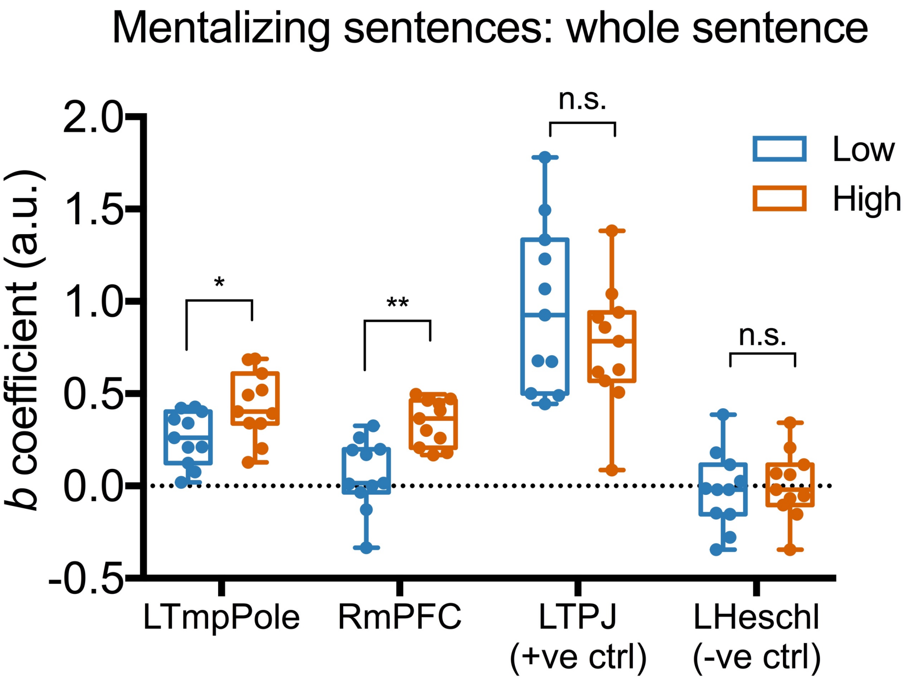

Supplementary Fig. 2. Group differences in response to mentalizing events based on a whole-sentence regressor. This is a reanalysis of the data shown in Fig. 5b; here, mentalizing events are modeled as the whole sentence containing an event (rather than just the sentence offset). See also *Creating the regressor* in the Methods section. Each dot represents a subject. Boxes represent the median and 25th/75th percentiles, and whiskers represent the minimum and maximum. *$p < 0.05$, **$p < 0.001$ (p-values adjusted to control the false discovery rate).
